# A Neural Conditional Random Field Model Using Deep Features and Learnable Functions for End-to-End MRI Prostate Zonal Segmentation

**DOI:** 10.59275/j.melba.2025-gc4c

**Published:** 2025-08-20

**Authors:** Alex Ling Yu Hung, Kai Zhao, Kaifeng Pang, Haoxin Zheng, Xiaoxi Du, Qi Miao, Demetri Terzopoulos, Kyunghyun Sung

**Affiliations:** 1 Computer Science Department, UCLA, Los Angeles, CA, USA; 2 Department of Radiological Sciences, UCLA, Los Angeles, CA, USA; 3 Electrical Engineering Department, UCLA, Los Angeles, CA, USA; 4 Bioengineering Department, UCLA, Los Angeles, CA, USA; 5 VoxelCloud, Inc., Los Angeles, CA, USA

**Keywords:** Graphical Models, Conditional Random Field, MRI, Prostate Zonal Segmentation

## Abstract

The automatic segmentation of prostate MRI often produces inconsistent performance because certain image slices are more difficult to segment than others. In this paper, we show that consistency can be improved using Conditional Random Fields (CRFs), which refine the segmentation results by considering pixel relationships pairwise. In practice, however, conventional CRFs are susceptible to noise and MRI intensity shifts due to their use of simple binary potentials involving spatial distance and intensity difference. Such heuristic potential functions are hardly expressive, limiting the network from extracting more relevant information and having more stable potential calculations. We propose a novel end-to-end Neural CRF (NCRF) model that utilizes learnable binary potential functions based on deep image features. Experiments show that our NCRF is a better model for prostate zonal segmentation than state-of-the-art CRF models. The NCRF improves segmentation accuracy in both the prostate transition zone and peripheral zone such that segmentation results are consistent across all the prostate slices, which can improve the performance of downstream tasks such as prostate cancer detection and segmentation. Our code is available at https://github.com/aL3x-O-o-Hung/NCRF.

## 1. Introduction

Magnetic Resonance Imaging (MRI) is favored for Prostate Cancer (PCa) diagnosis before biopsy because of its non-invasive nature (Appayya et al., 2018). According to the Prostate Imaging Reporting and Data System (PI-RADS v2.1) (Turkbey et al., 2019), variations in image appearance and cancer prevalence necessitate different analyses of PCa lesions in various prostate zones, specifically the Transition Zone (TZ) and the Peripheral Zone (PZ). Prostate zonal information is essential for the diagnosis of PCa, and accurate prostate zonal segmentation should be explicitly provided for accurate PCa lesion detection and segmentation. T2-weighted (T2w) MRI is the most common imaging modality for segmenting the prostate zones. Unfortunately, the manual annotation of prostate zones requires specialized expertise and is extremely time-consuming. Only experts can accurately delineate the prostate boundaries by carefully examining the correlations between pixels. Therefore, accurate automatic prostate zonal segmentation algorithms are needed to improve PCa diagnostics and treatment planning.

With the recent emergence of Deep Learning (DL) approaches, DL-based methods have become the dominant methods in medical image segmentation, progressively replacing traditional techniques (Bakator and Radosav, 2018; Litjens et al., 2017). U-Net (Ronneberger et al., 2015) and its variants, such as U-Net++ (Zhou et al., 2018) and UTNet (Gao et al., 2021), have become the foundational DL architectures for most state-of-the-art medical image segmentation methods across various different applications. For prostate segmentation, several DL-based methods have achieved impressive results (Bardis et al., 2021; Ronneberger et al., 2015; Liu et al., 2020; Zabihollahy et al., 2019; Aldoj et al., 2020), but they do not model the inter-pixel relationships, thus performing inconsistently across the different prostate slices (Nai et al., 2020; Hung et al., 2022).

To refine the segmentation results produced by DL-based methods by better modeling the relationships between pixels, Conditional Random fields (CRFs) (Lafferty et al., 2001), a class of discriminative undirected probabilistic graphical models that represent pixel labels as random variables forming a Markov Random Field (MRF) when conditioned on observations, have been employed as a post-processing step (Krähenbühl and Koltun, 2011; Kamnitsas et al., 2017; Dou et al., 2017; Dhawan et al., 2019). Others have proposed training the segmentation network alongside the CRF in an end-to-end manner, treating the CRF as a Recurrent Neural Network (RNN) (Zheng et al., 2015). By modeling the image as a CRF, where pixels serve as observed variables and labels are unobserved variables, better segmentation results can be achieved through the explicit modeling of the dependencies between predictions at different pixels via binary potentials (also termed “pairwise potentials” in the literature).

Typical CRFs, as is used in previous works (Zheng et al., 2015; Dhawan et al., 2019; Chen et al., 2022), whose binary potentials are based on pixel intensity similarities and spatial distance can, in theory, improve the overall performance and consistency of MRI prostate zonal segmentation; however, they can be problematic in practice. Due to the noisy nature, artifacts, and imaging characteristics of MRI, pixels from the same tissues or organs may exhibit considerably different pixel intensities (Krupa and Bekiesińska-Figatowska, 2015). Additionally, bias field signals can cause a certain region of a tissue or organ to appear darker than others (Guillemaud and Brady, 1997). On the other hand, prostate zonal segmentation can be particularly challenging in zones with relatively narrow morphology or ambiguous boundaries, where spatial-distance-based CRFs tend to over-smooth and lose structural detail. Moreover, current methods use predefined functions for binary potentials, limiting the CRF from more effectively modeling the relationships between pixels. Our method is designed to better handle such challenges by learning data-driven binary potentials, rather than relying on fixed assumptions about intensity or spatial proximity.

In this paper, we introduce a novel end-to-end Neural Conditional Random Field (NCRF) that uses no predefined functions or features for binary potentials; i.e., all the features and functions to calculate the potentials are learned. Using learnable functions and deep features to calculate the binary potentials, which is the main novelty of this work, enables the NCRF to autonomously determine what features and positional information are most relevant to calculating binary potentials while allowing more expressive functions to model the relationships between pixels. In this work, the NCRF is applied in 2D prostate MR image analysis, which is a unique application of CRF-based methods. The main contributions of our work are as follows:

1. We propose a novel end-to-end NCRF using both learnable functions and deep features to calculate binary potentials, enhancing the modeling of inter-pixel relationships.
2. We show that our NCRF achieves great 2D prostate zonal segmentation performance on *Internal Prostate* and *Prostate158* (Adams et al., 2022) as well as on whole prostate segmentation on *Promise12* (Litjens et al., 2014b), using as backbones both a fully convolutional network, nnU-Net (Isensee et al., 2018), and a transformer network, U-Net Transformer (Petit et al., 2021).
3. We show that our NCRF yields more consistent performance across all prostate parts—i.e., the apex, mid-gland, and base—by outperforming all competing methods in each of these regions in both TZ and PZ segmentation.
4. We demonstrate that the prostate zonal segmentation produced by our NCRF enables superior performance in downstream tasks, such as prostate cancer detection and segmentation, compared to competing methods.

## 2. Related Work

### 2.1 DL-Based Medical Image Segmentation

U-Net (Ronneberger et al., 2015) revolutionized medical image segmentation by employing an encoder-decoder Fully Convolutional Network (FCN) architecture. To further enhance segmentation performance, other researchers built upon U-Net, proposing similar encoder-decoder architectures, like U-Net++ (Zhou et al., 2018), nnU-Net (Isensee et al., 2018), and MSU-Net (Su et al., 2021). With attention mechanisms and transformer models (Vaswani et al., 2017) emerging in mainstream computer vision, medical image segmentation models have been proposed based on these architectures. MedT (Valanarasu et al., 2021) employed an axial attention mechanism in conjunction with a Local-Global (LoGo) training methodology. U-Net Transformer (Petit et al., 2021) incorporated self-attention and cross-attention mechanisms at the skip connections in U-Net. TNet (Gao et al., 2021) included not only attention modules but also full transformer blocks at the skip connections. CoTr (Xie et al., 2021) instead input the concatenation of all the feature maps from skip connections to a transformer block to produce the segmentation output. Swin Transformer (Liu et al., 2021) used a shift window between consecutive self-attention layers, rather than a fixed-size window like Vision Transformer (ViT) (Dosovitskiy et al., 2020). UNETR (Hatamizadeh et al., 2022) reformulated 3D segmentation as a 1D sequence-to-sequence prediction using a transformer model.

### 2.2 Image Segmentation With CRFs

The CRF was first proposed to segment and label sequence data with a graphical model (Lafferty et al., 2001). At first, most research focused on modeling the regional pixel or superpixel correlations with locally-connected CRFs (Shotton et al., 2009; Fulkerson et al., 2009; Triggs and Verbeek, 2007). To better model the global dependencies, Krähenbühl and Koltun (2011) considered fully connected CRFs defined on all the pixels in the image. As DL has increased in popularity, CRFs have also been widely adopted in medical image segmentation as a post-processing technique following an FCN in both natural image and medical image segmentation (Kamnitsas et al., 2017; Dou et al., 2017; Dhawan et al., 2019). Kamnitsas et al. (2017) used a fully connected CRF as a post-processing technique to their proposed dual pathway network for brain lesion segmentation. Dou et al. (2017) refined their segmentation output with a CRF. However, using the CRF as a post-processing technique does not allow end-to-end learning, limiting the network from adjusting its output to the CRF. Zheng et al. (2015) treated the CRF as an RNN, enabling end-to-end training of the entire CNN-CRF pipeline so that the network can better adapt to the CRF, resulting in improved overall performance. The same technique was applied in medical image segmentation and performed well on different organs and modalities (Xu et al., 2018; Thanh et al., 2021; Li et al., 2021). Although this has been shown to be effective in certain applications, these CRFs rely on spatial distance and differences in intensities between pixels. These heuristics make the method less generalizable to images from different patients and sources. Chen et al. (2022) proposed a posterior-CRF that, instead of using pixel intensities for the CRF binary potentials, makes use of deep features, increasing the robustness of the model.

### 2.3 MRI Prostate Zonal Segmentation

For MRI prostate zonal segmentation, Bardis et al. (2021) applied the U-Net to T2w images. Liu et al. (2020) enhanced DeepLabV3+ (Chen et al., 2018) with a Spatial Attentive Module (SAM) and modeled the epistemic uncertainty using dropout. Cuocolo et al. (2021) concluded that ENet (Paszke et al., 2016) outperforms U-Net and ERFNet (Romera et al., 2017) in MRI prostate zonal segmentation. Zabihollahy et al. (2019) segmented the TZ and PZ with two separate networks and refined the results with post-processing. Aldoj et al. (2020) came to the conclusion that Dense-2 U-Net was the best 2D DL model for prostate zonal segmentation on ProstateX dataset (Litjens et al., 2014a). Hung et al. (2022, 2024) used the information from other slices to guide the segmentation of the current slice via cross-slice attention mechanisms. Ren et al. (2023) applied a transformer encoder and decoder framework to MRI prostate zonal segmentation. CCT-U-Net (Yan et al., 2023), which was based on a convolution coupled transformer, was designed to retain edge details and better extract local features while capturing the long-term correlation between pixels for zonal segmentation.

## 3. Methods

### 3.1 Overview

**Figure 1:**
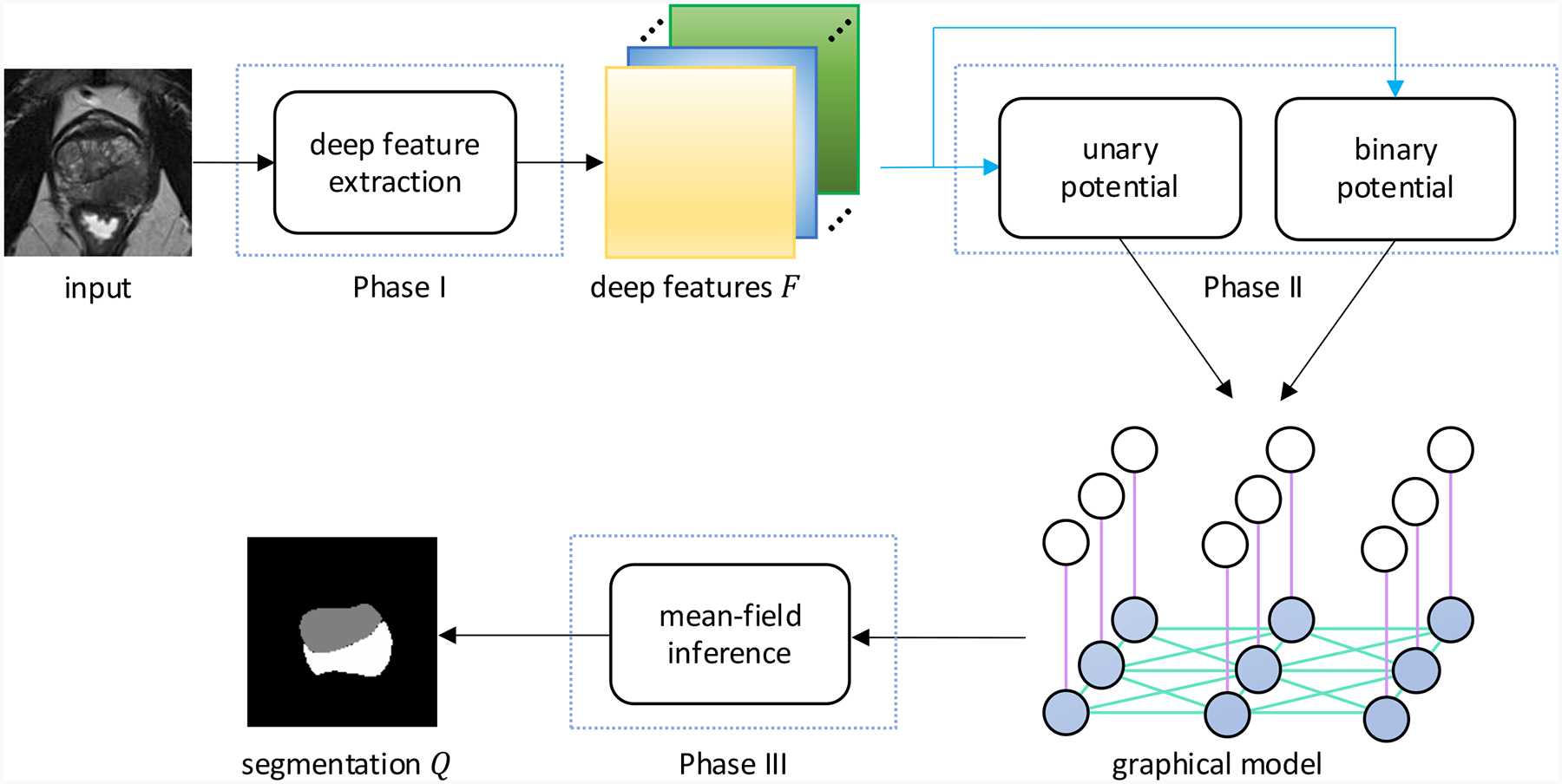
The NCRF pipeline. The shaded and unshaded nodes denote the labels and observations, respectively, while the purple and cyan lines denote unary and binary potentials, respectively, in the graphical model. The blue arrows indicate learnable functions. The deep features are extracted in Phase I. The deep features are then used to calculate the unary and binary potentials through learnable functions. Finally, the segmentation is computed using approximate mean-field inference.

Our method consists of three phases. In phase I, deep features $F\in {ℛ}^{H\times W\times C}$ are extracted from the input image I of height H and width W, where C is the number of features per pixel. In phase II, the F are used to compute both the unary potentials $\psi_{u}$ and binary potentials $\psi_{b}$ by learnable functions for the NCRF. In phase III, approximate mean-field inference (Jordan et al., 1999; Zheng et al., 2015) is utilized to calculate the predicted segmentation distribution Q based on the potentials. Fig. 1 illustrates the pipeline, and more details about the implementation of each phase are found in Section 3.3. The remainder of this section is organized as follows: Section 3.2 details how NCRFs work and how they differ from traditional CRFs; Section 3.3 describes the parameterization and implementation of NCRFs; Section 3.4 outlines how mean-field inference works on NCRFs.

### 3.2 Neural Conditional Random Field

CRFs are a class of discriminative undirected probabilistic graphical models that represent pixel labels as random variables forming a Markov Random Field (MRF) when conditioned on the observations. In image segmentation, the observations are typically the pixel intensities, but in our NCRF, they are the deep features F.

Let $X_{i,j}$, for 0≤i<H, 0≤j<W, be the random variable associated with pixel (i,j) representing the label, which can take any value from the label set ℒ={0,1,2,…,K-1}, where K is the number of classes. Given a graph G=(V,E) with set of nodes $V=\left\{X_{i,j}\right\}$ and set of edges E, as well as observations O, a CRF can be modeled by a Gibbs distribution

(1) $$P(X=x|O)=\frac{1}{Z(O)}\;\text{exp}(-ℰ(x|O)),$$

where Z(O) is the partition function and the Gibbs energy is

(2) $$ℰ(x|O)=\sum_{i,j}\psi_{u}\left(x_{i,j}|O\right)+\sum_{\left((i,j),\left(i^{\prime },j^{\prime }\right)\right)\in E}\psi_{b}\left(x_{i,j},x_{i^{\prime },j^{\prime }}|O\right).$$

Given observation O, the unary potentials $\psi_{u}\left(x_{i,j}|O\right)$ describe the cost of pixel (i,j) having label $x_{i,j}$ and the binary potentials $\psi_{b}\left(x_{i,j},x_{i^{\prime },j^{\prime }}|O\right)$ measure the cost of pixel (i,j) having label $x_{i,j}$ while pixel ($i^{\prime },j^{\prime }$) has label $x_{i^{\prime },j^{\prime }}$.

Traditionally, the CRF is constructed as a fully connected CRF, where edges exist between every pixel (Lafferty et al., 2001; Dhawan et al., 2019; Zheng et al., 2015). However, full connectivity, which significantly increases computational and memory demands, may not be necessary, as the labels of pixels that are far apart generally have minimal impact on each other. Any dependencies can typically be captured implicitly through the joint probability distribution. To balance expressiveness and efficiency, we construct our NCRF using only the 8 nearest neighbors for each pixel. This configuration captures both axis-aligned and diagonal spatial relationships, which are particularly important for delineating anatomical boundaries that may not follow strict horizontal or vertical orientations. The 8-connected neighborhood offers a compact yet expressive graph structure that effectively models local context while maintaining low computational cost.

Additionally, instead of using a predefined function to compute the binary potentials, in NCRFs we compute them as a set of learnable functions implemented by neural networks, parameterized as

(3) $$\psi_{b,\theta_{b}}\left(x_{i,j},x_{i^{\prime },j^{\prime }}|O\right)=f_{\theta_{b}}\left(x_{i,j},x_{i^{\prime },j^{\prime }},o_{i,j},o_{i^{\prime },j^{\prime }},(i,j),\left(i^{\prime },j^{\prime }\right)\right);$$

i.e., we treat the binary potentials as a function $f_{b,\theta_{b}}$ with learnable parameters $\theta_{b}$. This enables the network to learn more meaningful functions and deep feature representations. The unary potentials are also treated as a function with learnable parameters $\theta_{u}$:

(4) $$\psi_{u,\theta_{u}}\left(x_{i,j}|O\right)=f_{u,\theta_{u}}\left(x_{i,j},o_{i,j}\right).$$

### 3.3 Parameterization and Network Implementation

In Phase I, deep features $F\in {ℛ}^{H\times W\times C}$ are extracted from the input image I via a neural network $f_{\theta_{0}}$ parameterized by $\theta_{0}$, which is the backbone network, i.e. implemented as a nnU-Net (Isensee et al., 2018) and a U-Net Transformer (Petit et al., 2021) in this work. In Phase II, the deep features F are then fed into two separate functions $f_{u,\theta_{u}}$ and $f_{b,\theta_{b}}$ for unary and binary potentials, respectively, and we use a linear layer to compute the unary potential of each pixel from the corresponding deep features.

The deep features F are the observations O in computing the binary potentials. First, position information is incorporated into the function $f_{b,\theta_{b}}$ through positional encoding (Vaswani et al., 2017), which has been shown to be capable of informing deep networks of the positions of features (Hung et al., 2022; Dosovitskiy et al., 2020). This is important since CRFs need positional information for optimal performance Krähenbühl and Koltun (2011). We add the positional encoding $PE_{i,j}$ for pixel (i,j) to the observation $o_{i,j}$ at that location to obtain the positionally-encoded observation $o_{i,j}^{\prime }=o_{i,j}+PE_{i,j,c}$ with

(5) $$PE_{i,j,c}=\left\{\begin{array}{ll} \text{sin}(\omega j) & \text{when}\;c=4k,\\ \text{cos}(\omega j) & \text{when}\;c=4k+1,\\ \text{sin}(\omega i) & \text{when}\;c=4k+2,\\ \text{cos}(\omega i) & \text{when}\;c=4k+3, \end{array}\right.$$

where ω=exp(4klog(10000)/C),0≤k<C, and c is the index of the feature; i.e., the index of the channel. Next, a Multi-Layer Perceptron (MLP) $f_{\theta_{b,0}}$ is used to convert the new observation $o_{i,j}^{\prime }$ to new deep features

(6) $$\phi_{i,j}=f_{\theta_{b,0}}\left(o_{i,j}^{\prime }\right).$$

As these features contain both semantic and positional information, using separate features for semantic and positional information is unnecessary (cf., (Lafferty et al., 2001; Zheng et al., 2015; Chen et al., 2022)). Therefore, the binary potential between pixel (i,j) taking label $x_{i,j}$ and its neighbor ($i^{\prime },j^{\prime }$) taking label $x_{i^{\prime },j^{\prime }}$ is calculated as a function of only the classes $x_{i,j}$ and $x_{i^{\prime },j^{\prime }}$ as well as the new deep features $\phi_{i,j}$ and $\phi_{i^{\prime },j^{\prime }}$:

(7) $$\psi_{b}\left(x_{i,j},x_{i^{\prime },j^{\prime }}|O\right)=f_{\theta_{b,1}}\left(x_{i,j},x_{i^{\prime },j^{\prime }},\phi_{i,j},\phi_{i^{\prime },j^{\prime }}\right),$$

where

(8) $$f_{\theta_{b,1}}\left(x_{i,j},x_{i^{\prime },j^{\prime }},\phi_{i,j},\phi_{i^{\prime },j^{\prime }}\right)=M_{x_{i,j},x_{i^{\prime },j^{\prime }}},$$

is implemented as the exponential of another function $g_{\theta_{b,1}}$ which takes in the difference $d\left(\phi_{i,j},\phi_{i^{\prime },j^{\prime }}\right)$ between $\phi_{i,j}$ and $\phi_{i^{\prime },j^{\prime }}$ and generates K×K matrix

(9) $$M=\text{exp}\left(g_{\theta_{b,1}}\left(d\left(\phi_{i,j},\phi_{i^{\prime },j^{\prime }}\right)\right)\right)$$

representing the binary potentials between different classes.

### 3.4 Approximate Mean-Field Inference

After calculating the unary and binary potentials, an approximate mean-field inference process calculates the final output probabilities for each class. Algorithm 1, provides an overview of this process, where Q denotes the segmentation probability distribution that approximates the true distribution P, while N is the total number of iterations and Z is the partition function.

During the initialization step, we first initialize Q with the unary potentials $\psi_{u}$:

(10) $$Q\leftarrow \frac{1}{Z}\;\text{exp}\left(-\psi_{u}\right),$$

which is essentially equivalent to applying a softmax function over $-\psi_{b}$ across all the labels at every pixel.

During the message passing step, we compute an updated pseudo-potential $\tilde{\psi }$ based on the binary potential $\psi_{b}$ and Q:

(11) $$\overset{\tilde{\psi }\left(x_{i,j}|O\right)}{\leftarrow \sum_{\left((i,j),\left(i^{\prime },j^{\prime }\right)\right)\in E}}\sum_{0\leq x_{i^{\prime },j^{\prime }}<K}\psi_{b}\left(x_{i,j},x_{i^{\prime },j^{\prime }}|O\right)Q\left(x_{i^{\prime },j^{\prime }}\right),$$

where the compatibility of nearby labels and the probabilities of pixels being from certain classes at different locations are considered.

Finally, we update the approximate probability distribution

(12) $$Q\leftarrow \frac{1}{Z}\text{exp}(-\psi ),$$

by adding the unary potential $\psi_{u}$ to the pseudo-potential ψ:

(13) $$\psi \leftarrow \psi_{u}+\tilde{\psi }.$$

We apply these steps for N iterations to obtain the final output Q of the NCRF model.

## 4. Experiments

### 4.1 Datasets

#### 4.1.1 Internal Prostate

The images from this private dataset are acquired by 3-Tesla MRI scanners at a single academic institution. The dataset comprises 296 patients, with 238, 29, and 29 patients in the training, validation, and test sets, respectively. The dataset includes only T2w MR scans with an in-plane resolution of 0.625 mm^2^ and a through-plane resolution of 3 mm. The images are centrally cropped from 320 × 320 to 128 × 128. Clinical experts manually annotated the prostate TZ and PZ as ground truths.

#### 4.1.2 Prostate158

*Prostate158* (Adams et al., 2022) is a curated dataset of biparametric 3-Tesla prostate MRI. The slice thickness of the T2w images is 3 mm and the in-plane resolution is 0.47 mm. We resized the images from 270 × 270 to 180 × 180 and retained the center 128 × 128 for training and testing. The dataset is split into 139 images for training and 19 images for testing. The dataset was annotated by two different readers, and when annotations by both readers are available for a subject, we utilize the annotation by reader 1. For the downstream tasks of PCa detection and segmentation, we utilized T2w, Diffusion-Weighted Images (DWI), and Apparent Diffusion Coefficient (ADC) maps. PCa lesions were annotated in regions where the PI-RADS score was four or above.

**Algorithm 1:** NCRF approximate mean-field inference process

**Input:** unary potentials $\psi_{u}$ and binary potentials $\psi_{b}$	
**Output:** segmentation probability distribution Q	
l: $Q\leftarrow \frac{1}{Z}\;\text{exp}(-\psi_{u})$	▷ *initialize Q*
2: **for n←0** to N-1 **do**
3: **for** every position (i, j) **do**
4: **for** every possible label $x_{i,j}$ **do**
5: $\tilde{\psi }(x_{i,j}|O)\leftarrow \sum_{((i,j),(i\prime ,j\prime ))\in E}\sum_{0\leq x_{i\prime ,j\prime }<K}\psi_{b}(x_{i,j},x_{i\prime ,j\prime }|O)Q(x_{i\prime ,j\prime })$	▷ *message passing*
6: **end for**
7: **end for**
8: $\psi \leftarrow \psi_{u}+\tilde{\psi }$	▷ *add unary potential*
9: $Q\leftarrow \frac{1}{Z}\;\text{exp}(-\psi )$	▷ *update Q*
10: **end for**	

#### 4.1.3 Promise12

*Promise12* (Litjens et al., 2014b) is a T2w MR image dataset, comprising 80 MRI cases from 4 different imaging centers, with 50 cases for training and 30 cases for testing. We randomly select 10 cases as validation set and leave the rest 40 cases for model training. Whole-prostate segmentation annotation was performed by experienced readers, and all annotations were performed on a slice-by-slice basis.

### 4.2 Implementation Details and Evaluation Metrics

#### 4.2.1 Backbone Implementation

To show the versatility of our NCRF, we employed an FCN nnU-Net (Isensee et al., 2018) and a U-Net Transformer (Petit et al., 2021) as backbone networks. The base number of filters is 64 for all the networks, and the number of filters doubles at each layer. The decoder is the exact opposite of the encoder.

#### 4.2.2 State-of-the-Art Implementation

We compared the prostate zonal segmentation performance of our NCRF-based segmentation models with that of other popular methods; specifically, DeepLabV3+ (Chen et al., 2018), Liu et al.’s (Liu et al., 2020), nnU-Net (Isensee et al., 2018), U-Net Transformer (Petit et al., 2021), Zabihollahy et al.’s (Zabihollahy et al., 2019), CE-Net (Gu et al., 2019), MSU-Net (Su et al., 2021), and Dense-2 U-Net (Aldoj et al., 2020). We implemented the competing models per the specifications in the above cited original papers.

#### 4.2.3 Baseline Implementation

We also compared our NCRF segmentation models against other CRF-based segmentation models and a non-CRF-based segmentation baseline that is simply a naive backbone network. The first baseline, *postproc-CRF*, is a non-endto-end CRF-based model (Dhawan et al., 2019) in which the CRF serves to post-process the output of the backbone network. The second baseline, *spatial-CRF*, is an end-to-end CRF-based model whose binary potentials are based purely on spatial distance. The third baseline, *intensity-CRF*, is an end-to-end CRF-based model whose binary potentials are based on both spatial distance and pixel intensities (Zheng et al., 2015). The fourth CRF baseline, *posterior-CRF*, is an end-to-end CRF-based model with binary potentials based on spatial distance and posterior probabilities instead of pixel intensities (Chen et al., 2022).

#### 4.2.4 NCRF Implementation

We used the squared difference between feature maps as the difference function d(·) in (9), and we set the number of iterations in the mean-field inference (Algorithm 1) to N=5, as increasing N does not significantly improve results (Krähenbühl and Koltun, 2011).

#### 4.2.5 Training Details

We included all the slices from the patients in the training set during training, including slices without TZ and PZ. We used cross-entropy loss as the loss function in all the training procedures, and the Adam (Kingma and Ba, 2014) optimizer with a learning rate of 1 × 10^−4^ and weight decay regularization (Loshchilov and Hutter, 2018) with the parameter set to 1 × 10^−5^. For stable training, the back-propagating gradient is clipped at 0.01 during each step of the optimization. All the models were trained from scratch for 200 epochs and the best model was selected based on the validation set performance.

#### 4.2.6 Image Normalization

To provide inputs to the models, we normalized the images in the following ways. For the T2w images, the normalized intensity of pixel (i,j) on slice k is

(14) $${\tilde{I}}_{i,j,k}=\left(I_{i,j,k}-\mu \right)/\sigma ,$$

where $I_{i,j,k}$ is the un-normalized pixel intensity, and μ and σ are the per-patient pixel intensity mean and standard deviation, respectively. The DWI and ADC maps were rescaled to [0, 1] as follows:

(15) $${\tilde{I}}_{i,j,k}=\left(I_{i,j,k}-\text{min}(I)\right)/(\text{max}(I)-\text{min}(I)),$$

where max and min take the per-patient maximum and minimum pixel intensities.

#### 4.2.7 Data Augmentation

For the *Internal Prostate* dataset, only center crop, horizontal flip, and Gamma transform were performed. For the *Prostate158* and *Promise12* datasets, only center crop and horizontal flip were used. For a fair comparison, the data augmentation scheme was kept the same across all the experiments.

#### 4.2.8 Evaluation Metrics

To assess the segmentation results, we used the Dice Similarity Coefficient (DSC), Relative Absolute Volume Difference (RAVD), and Average Symmetric Surface Distance (ASSD), and all these metrics were calculated in a 3D per-patient manner. For experiments involve statistical testing, Wilcoxon Signed-Rank test is used. For evaluating the PCa detection performance, the local maxima on the output probability map were regarded as PCa detection points, defined as true positives (TP) if they are within 5 mm of any PCa ground truth pixels (Cao et al., 2019). The relationship between model detection sensitivity and the number of false positive (FP) predictions per patient was then analyzed.

### 4.3 Prostate Zonal Segmentation

We first compared the prostate zonal segmentation performance of our NCRF-based methods against other popular methods on both the *Internal Prostate* and *Prostate158* datasets. In these experiments, statistical test is also performed using Wilcoxon Signed-Rank test. Table 1 and Table 2 report the results, showing that the NCRF-based methods outperform the other popular methods. We note that *NCRF-nnU-Net* and *NCRF-U-Net Trans*. are NCRF-based methods with nnU-Net and U-Net Transformer as backbones, respectively. In the tables in this section, * and ^†^ indicate that *NCRF-nnU-Net* and *NCRF-U-Net Trans*. is better than the comparing method with statistical significance (p<0.05) respectively. Specifically, on *Internal Prostate*, *NCRF-nnU-Net* is almost always the best performer in both TZ and PZ segmentation, and *NCRF-U-Net Trans*. is the second best on TZ segmentation. On *Prostate158*, *NCRF-U-Net Trans*. is almost always the best performer in both TZ and PZ segmentation, while *NCRF-nnU-Net* is the second best performer. The effectiveness of our NCRF-based models is thus established by their outperformance of competing 2D prostate zonal segmentation models on two different datasets.

Furthermore, we compared our NCRF segmentation model on the *Internal Prostate* dataset using both nnU-net and U-Net Transformer as the backbone against existing CRF-based segmentation models and naive models lacking CRFs. Table 3 and Table 4 report the quantitative results. With nnU-Net as the backbone, our NCRF model achieves top performance in every metric in segmenting both the TZ and PZ. *spatial-CRF* performs decently in segmenting the TZ with the second-best performance in DSC and RAVD, but the performance in segmenting the PZ is not as good. Likewise, *posterior-CRF* has decent performance in segmenting the PZ, but its performance in segmenting the TZ is suboptimal. With the U-Net Transformer as the backbone, our NCRF model still has top performance in TZ segmentation in every metric while also having good performance in PZ segmentation overall. *intensity-CRF* appears to be the second best-performing method in TZ segmentation, and *non-CRF* is the best in PZ segmentation. Other methods are less consistent across different backbones and perform differently when segmenting different prostate zones, but our NCRF is consistently the top-performing method on *Internal Prostate* in the segmentation of different zones across different backbones.

We performed the same comparison on the *Prostate158* dataset, and the results are shown in Table 5 and Table 6. While using nnU-Net backbone, in segmenting the TZ, our NCRF model performs best while *posterior-CRF* has the second best. In segmenting the PZ, our NCRF model achieves the best DSC, third best RAVD, and second best ASSD, while *non-CRF* has the second best DSC and the best RAVD and ASSD. Although the NCRF model is not the clear best in segmenting the PZ, it is one of the top 2 methods. *posterior-CRF* achieves good results in segmenting the TZ, but it has subpar performance in segmenting the PZ. *Non-CRF* appears to have good performance in segmenting the PZ, but it is only the third best method in segmenting the TZ. When using the U-Net Transformer backbone, *NCRF* has top performance on both TZ and PZ, while *non-CRF* being the second. Compared with using nnU-Net backbone, the trends are similar in PZ segmentation with *NCRF* being the best in DSC, *non-CRF* being the best in RAVD, and both of them being comparable in ASSD. However, in TZ segmentation, *posterior-CRF* performs the second best while using nnU-Net but only the fourth while using U-Net Transformer. Our NCRF model is consistently one of the top 2 methods in segmenting both the TZ and PZ with both backbone networks on *Prostate158*.

**Figure 2:**
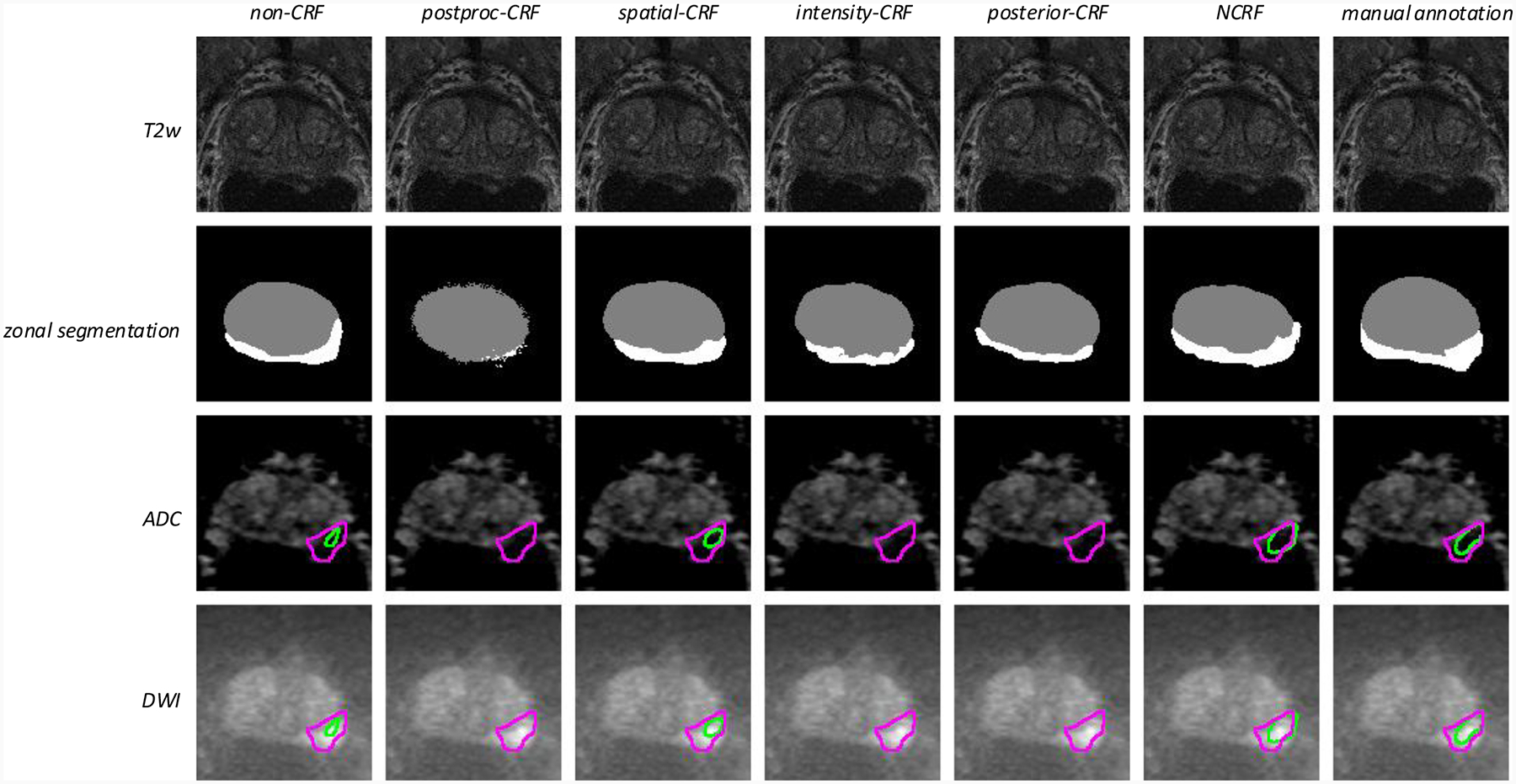
Qualitative NCRF segmentation results on the *Prostate158* dataset with the nnU-Net backbone. On the ADC maps and the DWI, purple demarks ground truth PCa lesions and green demarks the predicted PCa lesions. In the rightmost column of each case, the manual annotation is the ground truth zonal segmentation, while the result on the ADC map and the DWI is the PCa lesion segmentation with the ground truth zonal segmentation as input.

The rows of T2w images in Fig. 2 are the input images to be segmented from the *Prostate158* dataset, and in the second row, grey and white represent the TZ and PZ, respectively. Other methods fail to accurately delineate the shape of PZ.

### 4.4 Whole-Prostate Segmentation

The results of whole-prostate segmentation on *Promise12* using the nnU-Net and U-Net Transformer backbones are shown in Table 7. With the nnU-Net backbone, the NCRF model has the best DSC and the second best RAVD, while *intensity-CRF* has the second best DSC and *spatial-CRF* has the best RAVD. With the U-Net Transformer backbone, the NCRF model has the best performance in both metrics, while *intensity-CRF* performs well in DSC and *posterior-CRF* has the second best performance in RAVD. Our NCRF model consistently performs well in both metrics, while the other methods, including other CRF-based methods and non-CRF methods, do well only on a single metric. Although whole-prostate segmentation is a relatively simpler task than zonal segmentation and all competing methods perform decently, our NCRF model has the strongest performance using either of the two backbones, and it is consistently robust across the different datasets and backbones. The addition of the NCRF in the segmentation networks does improve the segmentation performance even on an easier task, i.e. whole-prostate segmentation.

### 4.5 Ablation Study

We performed an ablation study using the *Internal Prostate* dataset and the nnU-Net backbone. Table 8 reveals that using only the learnable function does not appreciably improve performance, whereas using both the learnable function and the positional encoding yields the best performance. These results suggest that the learnable function by itself cannot sufficiently calculate the binary potentials without positional information; i.e., the deep features alone do not contain enough information about the relative positions of the pixels. Combining the positional encoding and the learnable function yields the best performance, as better binary potentials can be calculated based on the deep features along with the explicit positional information.

### 4.6 Performance on Different Parts of the Prostate

**Figure 3:**
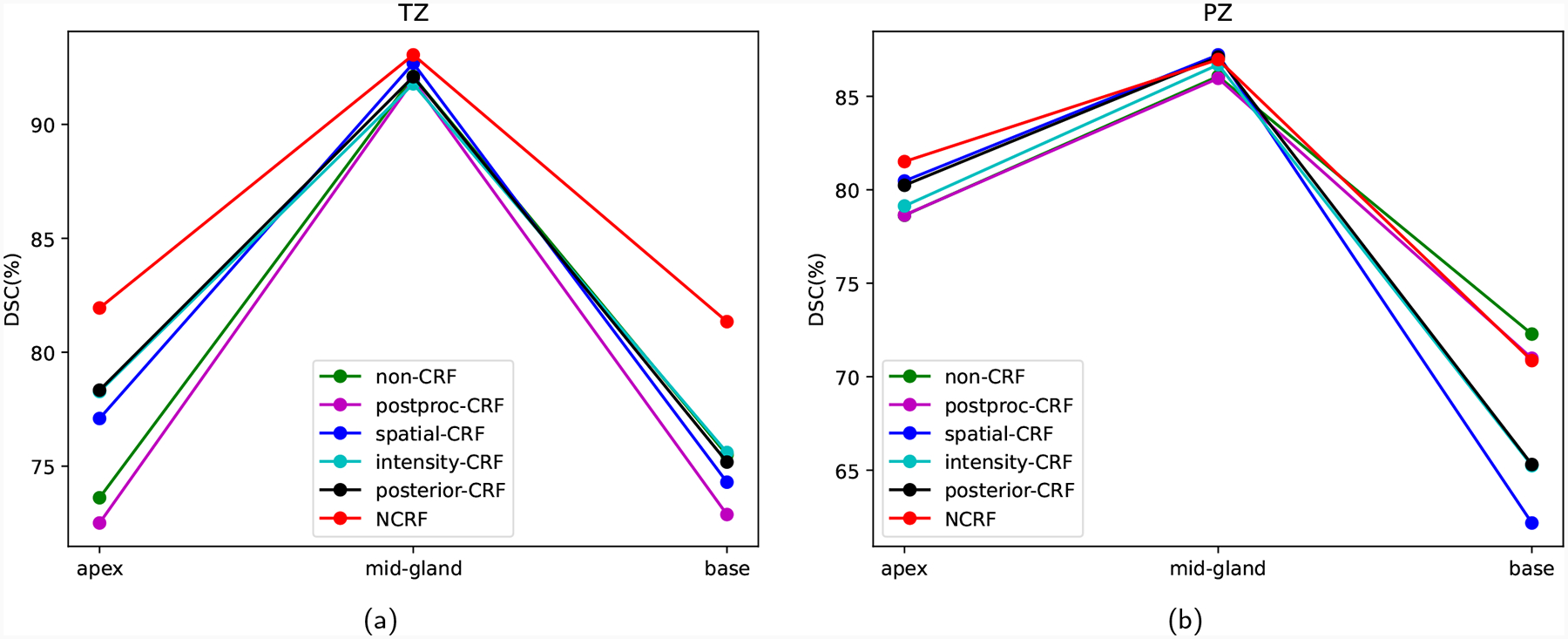
Comparison of (a) TZ and (b) PZ segmentation of different prostate parts on the *Internal Prostate* dataset.

DL models are notorious for inconsistent segmentation performance across all parts of the prostate (Hung et al., 2022). Using the *Internal Prostate* database and the nnU-Net backbone, we further compared our NCRF model against competing methods on all three parts of the prostate, with the convention that the first and last two image slices constitute the apex and base, respectively, and the remaining slices are mid-gland slices. Fig. 3 and Table 9 show that the NCRF model achieves the best TZ segmentation performance across the apex, mid-gland, and base, while being in the top-2 methods in PZ segmentation on all prostate parts. Although the *spatial-CRF* and *posterior-CRF* models have comparable TZ and PZ segmentation performance in the mid-gland, there is a larger gap between their performance and the performance of our NCRF model at the apex and base. Both the *spatial-CRF* and *posterior-CRF* models tend to have significantly worse performance in the base, even though they perform decently in the apex and mid-gland. Notably, *non-CRF* shows slightly higher PZ segmentation accuracy in the base, though the improvement is not statistically significant and comes at the cost of substantially lower performance in other regions. Although both apex and base regions contain thin anatomical structures, segmenting the PZ in base slices is more challenging due to increased anatomical variability, smaller PZ size, and weaker boundary contrast. The base of the prostate also interfaces with neighboring structures such as the bladder and seminal vesicles, which have similar intensity profiles in T2-weighted MRI and may obscure prostate boundaries. These factors likely reduce the effectiveness of spatial regularization methods in this region. Despite these challenges, NCRF maintains top-tier performance across all zones and regions, with particularly strong improvements in TZ segmentation in all slices and PZ segmentation in apex and mid-gland.

### 4.7 Downstream PCa Detection and Segmentation

**Figure 4:**
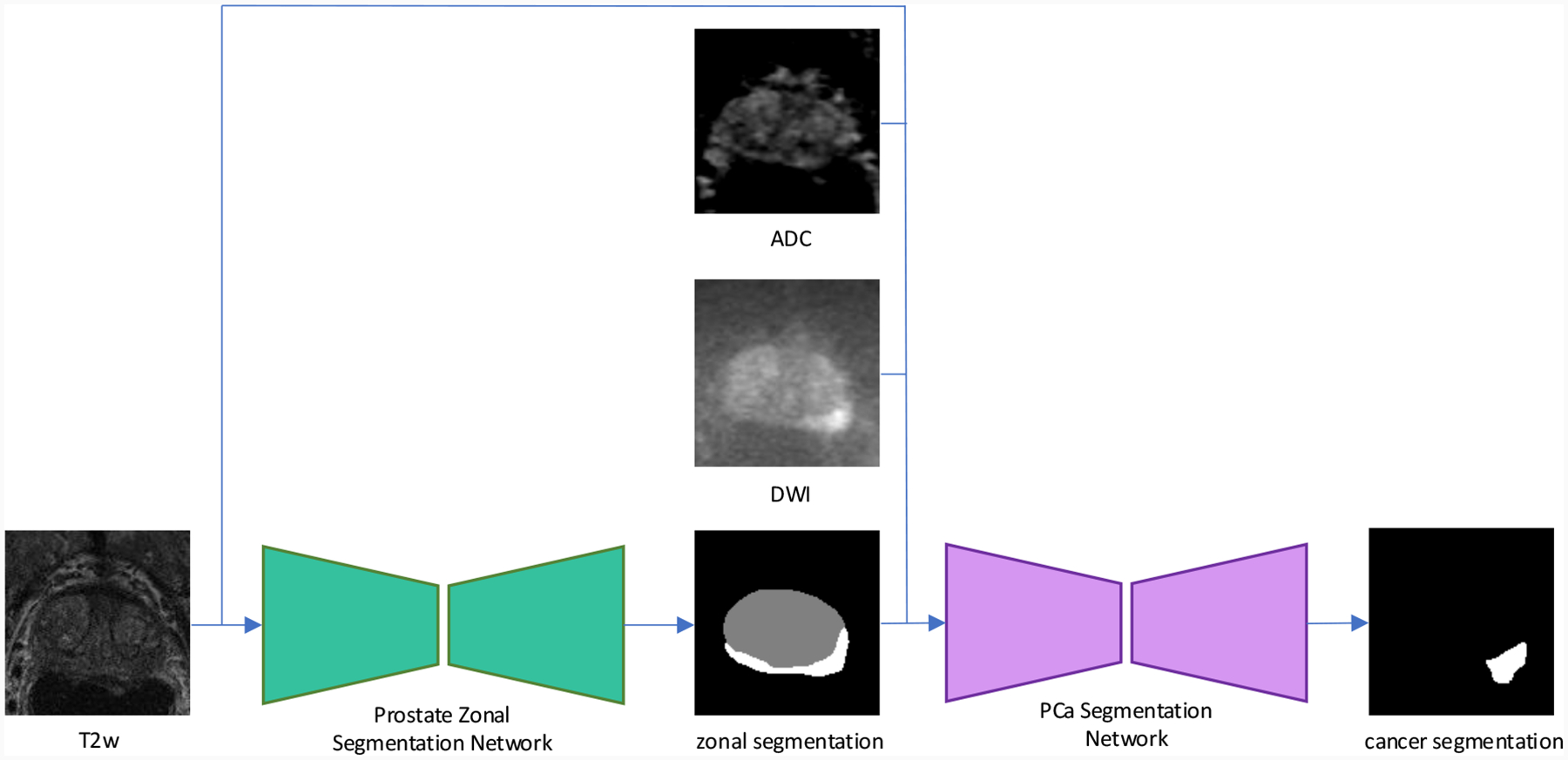
The pipeline of the cancer analysis experiment. The T2w image are first used as the input to the prostate zonal segmentation and then used as the input to the PCa segmentation network along with the ADC map, DWI and the zonal segmentation by the first network.

Using the *Prostate158* dataset, we further evaluated the zonal segmentation performance by utilizing the segmentation results to perform the downstream task of PCa detection and segmentation. A 2D nnU-Net was trained to perform the PCa analysis, with a 5-channel input of a T2w image, a DWI, and an ADC map, along with the segmentation of the TZ and PZ. During training, we use the ground truth TZ and PZ segmentation as input to the PCa analysis network. During inference, we utilized the prostate zonal segmentation results from different segmentation methods as input for the PCa analysis model, to evaluate the effectiveness of the prostate zonal segmentation produced by different models in the downstream task. Figure 4 shows the pipeline of this experiment on downstream cancer segmentation and detection. The backbone of all the CRF-based models was the nnU-Net. Table 10 shows that the PCa detection sensitivity using the zonal masks produced by the NCRF model is almost always the most accurate, even better than using the manual annotation in most cases. Furthermore, the PCa segmentation using the zonal masks produced by our NCRF model is much better than the competing models, while it is worse than the manual annotation of the prostate zones. This confirms the efficacy of our NCRF model in improving prostate zonal segmentation performance and its usefulness in downstream tasks.

Qualitative results are shown in Fig. 2. The first row shows the T2w images that are used as input to both the prostate zonal segmentation network and cancer analysis network. The second row shows the prostate zonal segmentation result, and the third and fourth rows show the ADC maps and DWI with the predicted and the ground truth lesion delineated. The green boundaries demark the predicted cancer lesions, and the purple boundaries indicate the ground truth. As the other models are unable to successfully differentiate between the TZ and PZ, their corresponding lesion segmentations are unsatisfactory. Only by inputting to the PCa segmentation model the zonal masks produced automatically by the NCRF model or manually can we get satisfactory PCa segmentation. This further demonstrates the capability and consistency of our NCRF model to perform accurate prostate zonal segmentation.

## 5. Discussion

Our comparison of NCRF-based segmentation models and other competing prostate zonal segmentation models shows that the former are generally the top performing models. In particular, according to Table 1 and Table 2 *NCRF-nnU-Net* performs better on the *Internal Prostate* dataset whereas *NCRF-Transformer U-Net* performs better on the *Prostate158* dataset. Although the U-Net Transformer and Zabihollahy et al.’s model perform respectably in TZ segmentation on both datasets, they are not as good in PZ segmentation. The NCRF-based models perform more consistently across both TZ and PZ segmentation.

Considering our experimental results when comparing NCRF with other CRF-based models in Table 3, Table 4 Table 5 and Table 6, *postproc-CRF* does not perform well on prostate image analysis in general, while every other competing method was at least the second-best approach on at least one occasion. Judging from the qualitative results, *postproc-CRF* tends to over-segment the predicted regions, especially in the PZ. The reason for this may be the noisy nature of MRI and the irregular shape of the prostate zones, especially the PZ, confuses the non-learnable post-processing since it is based purely on image intensity and spatial location. We have also observed that the other CRF-based models sometimes cannot outperform their CRF-lacking counterparts. The reason still stands if the images are extremely noisy and there is insufficient data from which the network can learn. Unlike other methods that rely on predefined functions to compute binary potentials—forcing the network to use them regardless of their relevance—our approach allows the network to learn these potentials adaptively. If the binary terms are too difficult or unnecessary to model for a given task, the network can effectively learn to ignore them. This may be why our method performs consistently well compared with the baseline methods.

Regarding the whole-prostate segmentation, shown in Table 7, all methods perform decently, and our NCRF model achieves only marginal improvement. Perhaps whole-prostate segmentation need not exploit many relationships pairwise to attain satisfactory accuracy, and the performance upper bound is determined by the ability of the backbone network. That said, our NCRF model still exhibits the best performance, meaning that even when such pixel relationships might not be needed, our approach would not hurt performance.

Our ablation study in Table 8 reveals that positional information is crucial for zonal segmentation and that our NCRF model can reach its full potential only when positional information is provided. Without it, the network cannot determine the position of each deep feature, resulting in suboptimal performance.

As is shown in Figure 2, our NCRF model consistently outperforms the other methods across different prostate parts, and by a larger margin in the more challenging apex and base regions. This is likely due to its improved ability to model pixel relationships pairwise, resulting in fewer false positive predictions in difficult slices.

The main limitation of the NCRF is its high memory consumption during training, restricting it to connections only to the 8 immediate neighbors for each pixel. Better memory optimization could enable denser graph connections, directly modeling longer-range pixel dependencies. Even though NCRF adds training and inference time to the model, the time added is not significant. As for training, it takes about 80 seconds for a normal nnU-Net and 110 seconds for *NCRF-nnU-Net* to train 1 epoch on *Internal*. To infer the zonal segmentation on *Internal*, it takes a normal nnU-Net 13 milliseconds per patient on average, while it takes *NCRF-nnU-Net* 20 milliseconds per patient.

Accurate and consistent automatic prostate zonal segmentation is crucial for the localization and staging of prostate cancer to enable MRI-targeted biopsy planning and guide for further therapy, including radiation, surgery, and focal ablation (Sonn et al., 2014). Our proposed NCRF improves the overall prostate zonal segmentation with improved consistency across all the prostate parts. This allows clinicians and downstream algorithms to make localizing prostate cancer easy and quick, without the need to manually annotate the prostate zones. The potential clinical impact of the proposed NCRF is confirmed by the experiment in Section 4.7, where the different segmentation methods are used in the downstream PCa detection and segmentation model. The results demonstrate how the improved prostate zonal segmentation by NCRF can boost the PCa detection and segmentation accuracy.

Although our proposed NCRF is currently applied within the scope of 2D prostate zonal segmentation, its design is inherently flexible and can be extended to 2.5D and 3D segmentation frameworks. The spatial message passing and learned pairwise potentials in NCRF, are not limited to 3D frameworks. For instance, in prostate zonal segmentation, where the resolution across slices is much poorer than that within slices, a 3D NCRF can be constructed by the 8 immediate neighbors within the slice and 2 immediate neighbors from nearby slices. In cases involving more isotropic volumes, a full 3D NCRF could incorporate all 26 immediate neighbors (6 face-connected, 12 edge-connected, and 8 corner-connected), the anatomical context and resolution. This potential extensibility could make NCRF a more versatile module for incorporating structured spatial relationships in a wide range of medical image segmentation applications beyond the 2D domain.

## 6. Conclusions

We have proposed a novel neural conditional random field model in which all the features and functions that contribute to calculating the binary potentials in the CRF are learned, thereby leveraging pairwise pixel relationships by learning the importance of features and positional information. Extensive experiments across three different prostate image datasets using two different backbone networks have demonstrated that our NCRF model can achieve tremendous performance under simple training settings without the need to tune the network extensively. Additionally, we have shown that the model performs more consistently across the apex, mid-gland, and base slices of the prostate. Our ablation study revealed that each component of the model contributes to its superior performance. Our experiments on downstream PCa detection and segmentation demonstrate how the improved prostate zonal segmentation by the model can be utilized in a clinical setting. Although our NCRF model was designed for prostate zonal segmentation in MRI, it can, in principle, be extended to be applicable to other organs and imaging modalities.

## Figures and Tables

**Table 1:** Our NCRF-based methods compared against other popular prostate zonal segmentation methods on *Internal Prostate*.

	TZ			PZ		
	DSC(%)↑	RAVD(%)↓	ASSD(mm)↓	DSC(%)↑	RAVD(%)↓	ASSD (mm)↓
DeepLabV3+	87.8*^†^	24.2*^†^	0.285*^†^	79.9*^†^	38.1*^†^	0.479*^†^
Liu et al.’s	87.3*^†^	25.6*^†^	0.292*^†^	81.3*^†^	35.9*^†^	0.455*^†^
nnU-Net	88.7*^†^	22.4*^†^	0.215*	83.4*	34.2*	0.347
U-Net Transformer	89.4^†^	21.2^†^	0.218	84.0	30.8	0.462
Zabihollahy et al.’s	89.1*^†^	21.6*^†^	0.227	81.4*	38.4*	0.640*^†^
CE-Net	87.5*	25.3*	0.253*^†^	80.7*	36.1*	0.524*
MSU-Net	88.6*^†^	22.4*^†^	0.214*	82.3*	34.1*	0.431*
Dense-2 U-Net	89.2*	22.1*^†^	0.217*	82.8*^†^	32.7*^†^	0.252
*NCRF-nnU-Net*	90.4	19.4	0.186	85.0	29.6	0.313
*NCRF-U-Net Trans*.	90.1	19.8	0.187	83.8	32.8	0.453

Red and blue in this and subsequent tables indicate the best and second best results, respectively. For this and the next table, * indicates statistical significance (p<0.05) when comparing against *NCRF-nnU-Net*, and † indicates statistical significance when comparing against *NCRF-U-Net Trans*.

**Table 2:** Our NCRF-based methods compared against other popular prostate zonal segmentation methods on *Prostate158*.

	TZ			PZ		
	DSC(%)↑	RAVD(%)↓	ASSD(mm)↓	DSC(%)↑	RAVD(%)↓	ASSD(mm)↓
DeepLabV3+	87.9*^†^	24.6*^†^	0.299*^†^	72.4*^†^	54.9*^†^	0.781*^†^
Liu et al.’s	85.9*^†^	28.5*^†^	0.383*^†^	71.5*^†^	53.1*^†^	0.713*^†^
nnU-Net	87.9^†^	24.1^†^	0.279	76.1^†^	45.1^†^	0.534
U-Net Transformer	89.3 ^†^	20.6 ^†^	0.311*	76.7^†^	43.4 ^†^	0.550
Zabihollahy et al.’s	89.0*^†^	22.0*^†^	0.263*	73.8*^†^	50.3^†^	0.830*^†^
CE-Net	87.0*^†^	25.1*^†^	0.352*^†^	71.8*^†^	52.5*^†^	0.695^†^
MSU-Net	87.7*^†^	23.8*^†^	0.306*^†^	72.8*^†^	45.7^†^	0.606
Dense-2 U-Net	86.0*^†^	29.2*^†^	0.364*^†^	65.2*^†^	56.8*^†^	1.038*^†^
*NCRF-nnU-Net*	88.4	22.9	0.223	77.2	47.5	0.557
*NCRF-U-Net Trans*.	89.6	20.5	0.223	77.2	44.5	0.533

**Table 3:** NCRF compared against the baseline methods on the *Internal Prostate* dataset using the nnU-Net backbone.

	TZ			PZ		
	DSC(%)↑	RAVD(%)↓	ASSD(mm)↓	DSC(%)↑	RAVD(%)↓	ASSD(mm)↓
*non-CRF*	88.7	22.4	0.215	83.4	34.2	0.347
*postproc-CRF*	88.4	22.8	0.213	83.4	33.1	0.323
*spatial-CRF*	89.1	21.9	0.222	84.4	31.6	0.349
*intensity-CRF*	88.5	22.9	0.232	83.9	32.2	0.328
*posterior-CRF*	88.8	23.8	0.224	84.6	31.4	0.302
*NCRF*	90.4	19.4	0.186	85.0	29.6	0.313

**Table 4:** NCRF compared against the baseline methods on the *Internal Prostate* dataset using the U-Net Transformer backbone.

	TZ			PZ		
	DSC(%)↑	RAVD(%)↓	ASSD(mm)↓	DSC(%)↑	RAVD(%)↓	ASSD(mm)↓
*non-CRF*	89.4	21.2	0.218	84.0	30.8	0.462
*postproc-CRF*	85.6	27.9	0.240	77.2	40.2	0.508
*spatial-CRF*	89.3	21.5	0.223	83.2	34.4	0.497
*intensity-CRF*	89.6	21.1	0.203	83.2	31.0	0.328
*posterior-CRF*	89.3	21.9	0.219	83.1	33.4	0.514
*NCRF*	90.1	19.8	0.187	83.8	32.8	0.453

**Table 5:** NCRF compared against the baseline methods on the *Prostate158* dataset using the nnU-Net backbone.

	TZ			PZ		
	DSC(%)↑	RAVD(%)↓	ASSD(mm)↓	DSC(%)↑	RAVD(%)↓	ASSD(mm)↓
*non-CRF*	87.9	24.1	0.279	76.1	45.1	0.534
*postproc-CRF*	84.4	30.8	0.366	65.7	55.0	0.792
*spatial-CRF*	87.7	25.2	0.244	75.7	46.5	0.573
*intensity-CRF*	87.1	25.8	0.277	72.7	51.5	0.694
*posterior-CRF*	88.2	23.7	0.227	74.6	47.8	0.606
*NCRF*	88.4	22.9	0.223	77.2	47.5	0.557

**Table 6:** NCRF compared against the baseline methods on the *Prostate158* dataset using the U-Net Transformer backbone.

	TZ			PZ		
	DSC(%)↑	RAVD(%)↓	ASSD(mm)↓	DSC(%)↑	RAVD(%)↓	ASSD(mm)↓
*non-CRF*	89.3	20.6	0.311	76.7	43.4	0.550
*postproc-CRF*	86.1	26.9	0.311	68.1	52.7	0.735
*spatial-CRF*	88.5	22.0	0.272	75.4	44.1	0.553
*intensity-CRF*	89.0	21.6	0.249	75.8	44.6	0.556
*posterior-CRF*	88.6	22.4	0.278	73.5	47.0	0.611
*NCRF*	89.6	20.5	0.223	77.2	44.5	0.533

**Table 7:** Comparison of the NCRF model against the baseline models on the *Promise12* dataset for whole prostate segmentation with nnU-Net and U-Net Transformer as backbones.

	nnU-Net		U-Net Transformer	
	DSC(%)↑	RAVD(%)↓	DSC(%)↑	RAVD(%)↓
*non-CRF*	87.8	25.0	88.3	23.9
*postproc-CRF*	86.3	26.1	83.0	30.7
*spatial-CRF*	87.9	24.3	88.1	24.5
*intensity-CRF*	88.0	24.8	88.4	24.0
*posterior-CRF*	87.8	24.8	88.3	23.7
*NCRF*	88.1	24.6	88.4	23.6

**Table 8:** Ablation study using the *Internal Prostate* dataset and the nnU-Net backbone, where PE and LF denote positional encoding and learnable function, respectively.

		TZ			PZ		
PE	LF	DSC(%)↑	RAVD(%)↓	ASSD(mm)↓	DSC(%)↑	RAVD(%)↓	ASSD(mm)↓
		88.7	22.4	0.215	83.4	34.2	0.347
✓		89.6	21.2	0.197	84.4	31.5	0.326
	✓	87.5	22.3	0.228	83.6	33.6	0.361
✓	✓	90.4	19.4	0.186	85.0	29.6	0.313

**Table 9:** DSC (%) comparison of the NCRF model against other CRF-based models on *Internal Prostate* dataset for prostate zonal segmentation on different prostate parts.

	TZ			PZ		
	apex	mid-gland	base	apex	mid-gland	base
*non-CRF*	73.6*	92.1*	75.5	78.6*	86.1*	72.3
*postproc-CRF*	72.5*	91.9*	72.9*	78.6*	86.0*	71.0
*spatial-CRF*	77.1*	92.7	74.3*	80.5 *	87.2	62.2
*intensity-CRF*	78.3	91.8*	75.6 *	79.1*	86.7	65.2
*posterior-CRF*	78.3 *	92.1*	75.2*	80.2	87.1	65.3
*NCRF*	81.9	93.1	81.3	81.5	87.0	71.0

* indicates statistical significance (p<0.05) when comparing against *NCRF*.

**Table 10:** Results of downstream PCa detection and segmentation using zonal masks produced by the different models.

	PCa Detection						PCa Segmentation
	Sensitivity @ FP/Patient(%)↑						DSC(%)↑
	0.5	1	1.5	2	2.5	3	
*non-CRF*	42.9	66.7	76.2	76.2	76.2	81.0	45.1
*postproc-CRF*	38.1	52.4	61.9	71.4	71.4	71.4	41.6
*spatial-CRF*	23.8	71.4	76.2	81.0	81.0	81.0	45.1
*intensity-CRF*	47.6	62.0	71.4	71.4	76.2	76.2	37.7
*posterior-CRF*	23.8	47.6	66.7	80.9	80.9	85.7	41.7
*NCRF*	38.1	71.4	76.2	81.0	85.7	85.7	50.2
*manual annotation*	52.4	71.4	71.4	76.2	81.0	81.0	54.8

## Data Availability

*Prostate158* is publicly available at https://github.com/kbressem/prostate158 and and *Promise12* is available at https://promise12.grand-challenge.org/. Our private dataset, *Internal Prostate* may be made available upon request in compliance with institutional IRB requirements.
